# Supplementary material for: COVID-19 Vaccine and Social Media in the U.S.: Exploring Emotions and Discussions on Twitter
Source: Vaccines (Basel). 2021 Sep 23;9(10):1059. doi: 10.3390/vaccines9101059 (PMC8540945; doi:10.3390/vaccines9101059)
Supplement: Supplementary file 1 [file vaccines-09-01059-s001.zip › vaccines-1274648-supplementary.pdf]

Supplementary Table S1. Topics and Examples of Tweets. Products and people were replaced by [].

ID	Topic	Example
T1	Vaccine Exemption Bill	Colorado [] wrote legislation that would put Americans in forced reeducation simply for refusing a vaccine including untested DNA altering vaccines. No media outlet covered it no politicians condemned the legislation Easy to verify
T2	Vaccine Distribution	We can't deliver administer vaccines efficiently effectively nationwide either We never learn what to do as a fed action decentralized or privatized Pandemic was a national emergency needed a national vaccine rollout like a cat 5 hurricane hitting everywhere
T3	Death and Vaccine	Miami doctor dies within weeks of receiving [] Covid19 vaccine Miami obstetrician Gregory Michael 58 died after a catastrophic reaction to the [] Covid19 vaccine
T4	Vaccine Information Sharing	Need fair balance information on safety C19 vax safety Doctors should weigh decision with patients on risks and benefits C19 recovered children pregnant women lowrisk should defer
T5	Politician Hoax	Its a hoax [] rushes to first in line for vaccine Selfish selfcentered hypocrite [] [] Coward
T6	Vaccination Sites	Monday s vaccination appointments at Alamodome WellMed clinics rescheduled due to expected wintry weather in San Antonio KSAT San Antonio
T7	Vaccination Hesitancy	The problem with saying this is it makes people feel the vaccine doesn't matter If you want people to get and trust the vaccine saying nothing will change if you get it or not isn't going to help in that cause
T8	Emergency Approval of Vaccines	FDA Panel Says [] PFE Vaccine s Benefits Outweigh Risks A panel for FDA advisers voted in favor of [] and [] s covid19 vaccine on grounds that its benefits outweigh risks. The vote was 17 to 4 with one abstention
T9	Vaccines' Mechanism	My understanding is the [] vaccine is not a weakened virus. It is the mRNA of the virus. This mRNA enters the cells and then replicates another component of the virus the spike protein Antibodies are produced to attack these spike proteins in future exposures
T10	Vaccine for Teachers	Teachers should be in a priority group for the vaccine. Virtual learning is not working for the students. I work in college admissions. These kids are not performing academically. Teachers tell me this
T11	Vaccination, Mask, and Social Distancing	My masking does not protect me it protects you from me and your masking protects me from you. Please wash your hands wear a mask stay home if sick and get the vaccine if you can Thank you
T12	Vaccine Immunity	Some early evidence suggests natural immunity may not last very long. We wont know how long immunity produced by vaccination lasts until we have more data on how well the vaccines work
T13	Vaccine Effectiveness	South Africa is suspending its rollout of the [] vaccine after initial trials showed disappointing results against the B1351 variant of COVID19

T14	Friends and Family Vaccination	The nursing home called me today to ask if I wanted my mother to get the COVID vaccine. Oh please please please give it to her as soon as you can. I told them I haven't hugged my mother since March. She's 96yrs old and I don t know how much time I have left with her
T15	Vaccination and Election	[] voters rewarded Pres [] successful warp speed Vaccine program by kicking him out after 2 attempted coups by the corrupt [] the Russian Hoax and false impeachment. Finally the cheating rigged election has done the job. The fake media lies and coverup did its dirty deed
T16	Trump Administration Performance	The response from the [] Administration was so pathetic it cost thousands of Americans their lives [] going down in the history books as the KILLER PRESIDENT. He thought he'd come rushing in on his white horse and save the day with a vaccine 330 thousand dead Americans
T17	Vaccine Development Timeline	We knew it was coming but we didn't know it was going to be this year. We all though maybe next year or years from now as it usually takes 45 years to create a vaccine and then get it approved. At the start of the pandemic we never thought to see a vaccine this soon
T18	Vaccination for Health Workers and Nursing Home Residents	Health care workers and nursing home residents should be at the front of the line when the first coronavirus vaccine shots become available an influential government advisory panel said Tuesday
T19	Vaccine Management	FDA says [] vaccine vials hold extra doses expanding supply Using every drop from overfilled vials could boost available doses by up to 40 percent
T20	Travel Mandatory Testing and Vaccine	Beginning Nov 24 anyone flying to Hawaii will have to show a negative COVID19 test before their departure no matter the airline they fly in onwhile [] airline [] will require proof of vaccination to fly
T21	Getting Vaccines Stories	I got my appoint today for 2nd dose thru email from HHD COVID19 Vaccine 2nd Dose Confirmation Good news for all those waiting on appointment for 2nd dose
T22	Vaccination Impact on Market	Markets have spent November celebrating upbeat vaccine news and closure on US election uncertainty After a strong month are equities headed for another reset
T23	Biden vs. Trump on Vaccine	[] scientific advisers will meet with vaccine makers in coming days as the presidential transition remains stalled because of President []'s refusal to acknowledge that he lost the election
T25	Supporting Pharmaceutical Companies	He signed the national vaccine injury compensation bill that exempts vaccine makers from liability for childhood vaccinations No surprise there
T26	Vaccine Side Effects	Update on my second dose of the [] vaccine. Got vaccinated at 12ish yesterday and felt achy and drained by the evening. Took some Ibuprofen and actually felt better before bed. Today felt completely normal other than a sore arm where I received the shot Feeling lucky
